# Comment to “COVID-19 vaccination, all-cause mortality, and hospitalization for cancer: 30-month cohort study in an Italian province”

**DOI:** 10.17179/excli2025-8974

**Published:** 2025-12-18

**Authors:** Giovanni M. Malatesta, Marco Alessandria, Franco Berrino, Alberto Donzelli

**Affiliations:** 1 Scientific Committee of the Foundation "Allineare Sanità e Salute", 51100 Pistoia, Italy; 2 Department of Life Sciences and Systems Biology, University of Turin, 10123 Turin, Italy; 3 Department of Theoretical and Applied Sciences, eCampus University, 22060 Novedrate, Italy; 4 (past) Department of Predictive and Preventive Medicine, Fondazione IRCCS Istituto Nazionale dei Tumori, 20133 Milan, Italy; 5 Independent Medical-Scientific Commission, Foundation "Allineare Sanità e Salute", 20131 Milan, Italy

## ⁯⁯

We read with interest the study recently published in your journal, "COVID-19 VACCINATION, ALL-CAUSE MORTALITY, AND HOSPITALIZATION FOR CANCER: A 30-MONTH COHORT STUDY IN AN ITALIAN PROVINCE" (Acuti Martellucci et al., 2025[1]). It is the first epidemiological study trying to link COVID-19 vaccination with the onset of neoplastic diseases. After several case reports and studies investigating possible biological mechanisms, we welcome a real-world study investigating the association between COVID-19 vaccination and cancer, by comparing vaccinated and unvaccinated individuals in a population large enough to obtain statistically significant results. For the first time, a statistically significant hazard ratio (HR) has been found for hospitalizations for all cancers among vaccinated individuals compared to unvaccinated, as well as for individual cancers (colorectal, breast, bladder). Statistical significance was not achieved for other cancers, but, among the nine cancers investigated, only one had an HR ˂ 1 (lung cancer, keeping in mind a higher smokers prevalence in unvaccinated: Ebrahimi Kalan et al., 2023[4]) and another (prostate cancer) close to 1.

However, the increase in tumors found by the authors could also be due to factors unrelated to actual increases, which should be further investigated based on the original data.

Anyway, the study also investigates the possible relationship between COVID-19 vaccination and the risk of all-cause deaths, finding a result deemed implausible by the authors themselves. Indeed, they find that COVID-19 vaccination was associated with lower all-cause deaths, far beyond what would be expected from protection from COVID-19 deaths: an HR 0.42 for the vaccinated with at least 1 dose compared to unvaccinated. The authors identify the healthy-vaccinee bias as the main explanation for this result. We agree with the presence of a healthy-vaccinee bias (which has proven to be larger and more persistent than the authors and others believe: Jackson et al., 2006[6]), but this is not the only bias in this study, as well as in previous studies on this population.

In fact, a previous study on the same province by the same research group (Flacco et al., 2022[5]) showed an even less plausible HR of all-cause death for vaccinated with at least one dose compared to unvaccinated: 0.19. In another article (Berrino et al., 2023[3]), we showed the presence of another bias fairly common in observational studies: the Immortal Time Bias (ITB). It consists in not considering, in the calculation of person-times, the time during which the vaccinated had not yet received the first dose, when they were still unvaccinated.

Even in a subsequent article by the same research group (Rosso et al., 2023[7]), concerning the same population, the ITB was evident. When the analysis of the all-cause mortality among the vaccinated compared with the unvaccinated was stratified according to the number of doses, the resulting HRs were contradictory: for the first and second doses the HRs were significantly > 1, while for the recipients of ≥ 3 doses the HR was 0.22, once again implausible and significantly ˂ 1. We therefore asked the authors for the dataset used for their analysis. Once received, we realigned the data to a single index date, to correct the ITB. The results of our analysis were published in a new article (Alessandria et al., 2024[2]), and showed that all HRs were higher than those found in (Rosso et al., 2023[7]). For those vaccinated with ≥ 3 doses, the HR was very close to 1 with no significant difference with respect to that of the unvaccinated. However, estimating the Restricted Mean Time Lost (RMTL), there was a small, but significant, greater loss of life expectancy for vaccinated with both 2 and ≥ 3 doses compared to the unvaccinated.

Now, the new study (Acuti Martellucci et al., 2025[1], see Table 1(Tab. 1)) has the same design as previous studies in the journal *Vaccines*, but still ignores the ITB. Only those not receiving any vaccine dose (never vaccinated) were considered 'unvaccinated'; instead, also the vaccinated were 'unvaccinated' before receiving the first dose. We do not have the individual data for now, but, using the same methodology as in our earlier contribution (Berrino et al., 2023[3]), we can roughly estimate the correct person-times for the unvaccinated, based on the average follow-up times published in the study.

This is a rough estimate, not adjusted for various confounding factors. Nevertheless, without considering the so-called immortal time (the time elapsed by vaccinated people before receiving the first dose, during which they were unvaccinated, but also evidently not dead), the crude all-cause mortality rate for the unvaccinated appears almost double than that of the vaccinated (1.21 vs 0.67). Instead, correcting for the unvaccinated person-times, the unvaccinated death rate is lower than that of the vaccinated (0.58 vs 0.67).

So, the implausible protection from the risk of all-cause deaths by COVID-19 vaccination resulting from the article is not attributable only to the healthy-vaccinee bias, but also to the ITB.

We kindly ask the authors for the raw data, to allow for a double re-analysis and a fruitful scientific discussion.

## Declaration

### Conflict of interest

The authors have nothing to declare.

### Artificial Intelligence (AI) - assisted technology

We did not use artificial intelligence.

## Figures and Tables

**Table 1:**
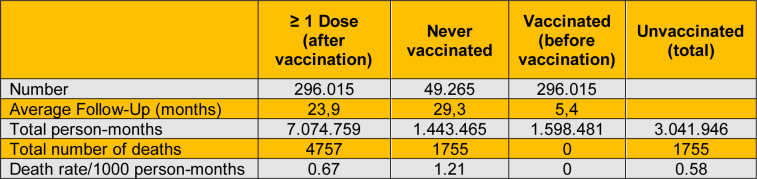
Recalculation of the estimates of numbers, average follow-ups, person-months, total deaths, and death rate of subjects with ≥ 1 vaccine dose versus unvaccinated in the study of Acuti Martellucci et al. (2025)
